# Simplified activity cliff network representations with high interpretability and immediate access to SAR information

**DOI:** 10.1007/s10822-020-00319-9

**Published:** 2020-06-05

**Authors:** Huabin Hu, Jürgen Bajorath

**Affiliations:** Department of Life Science Informatics, B-IT, LIMES Program Unit Chemical Biology and Medicinal Chemistry, Rheinische Friedrich-Wilhelms-Universität, Endenicher Allee 19c, 53115 Bonn, Germany

**Keywords:** Activity cliffs, Reduced activity cliff networks, SAR information, Matching molecular series, R-group tables

## Abstract

Activity cliffs (ACs) consist of structurally similar compounds with a large difference in potency against their target. Accordingly, ACs introduce discontinuity in structure-activity relationships (SARs) and are a prime source of SAR information. In compound data sets, the vast majority of ACs are formed by differently sized groups of structurally similar compounds with large potency variations. As a consequence, many of these compounds participate in multiple ACs. This coordinated formation of ACs increases their SAR information content compared to ACs considered as individual compound pairs, but complicates AC analysis. In network representations, coordinated ACs give rise to clusters of varying size and topology, which can be interactively and computationally analyzed. While AC networks are indispensable tools to study coordinated ACs, they become difficult to navigate and interpret in the presence of clusters of increasing size and complex topologies. Herein, we introduce reduced network representations that transform AC networks into an easily interpretable format from which SAR information in the form of R-group tables can be readily obtained. The simplified network variant greatly improves the interpretability of large and complex AC networks and substantially supports SAR exploration.

**Electronic supplementary material:**

The online version of this article (doi:10.1007/s10822-020-00319-9) contains supplementary material, which is available to authorized users.

## Introduction

Activity cliffs (ACs) are generally defined as pairs or groups of structurally similar or analogous compounds that share the same biological activity but have large differences in potency [1–3]. Accordingly, ACs encode small chemical changes having large effects on compound potency, which rationalizes their relevance for structure-activity relationship (SAR) analysis and chemical optimization [1–6]. For AC assessment, it must be decided when two compounds are sufficiently similar and their potency differences large enough to qualify as an AC. The evaluation of molecular similarity depends on chosen molecular representations and similarity measures [7]. For AC definition, different similarity and potency difference criteria are applicable and their choice characterizes different generations of ACs [8]. For systematic computational identification and analysis of ACs, consistent definitions must be applied [2, 3]. In addition, reliable AC assignments also depend on the use of high-quality activity measurements [6]. Much of our current knowledge about ACs and their distribution has resulted from systematic search calculations in large compound databases. Depending on the molecular representations that are used for structural similarity assessment and potency difference criteria that are applied, the frequency of ACs moderately varies. For example, ~ 20–30% of bioactive compounds participate in the formation of ACs and ~ 5–6% of pairs of structurally similar compounds form ACs if an at least 100-fold difference in potency is required [2, 3]. When alternative AC definitions are considered in parallel, on the order of 100,000 ACs are obtained on the basis of currently available bioactive compounds (unpublished data), which provide a rich source of SAR information.

One of the most important characteristics of ACs is that they rarely represent “isolated” compound pairs, i.e., compounds having no other structural neighbors. Instead, ACs are typically formed by groups of structurally similar compounds with significant potency variations, giving rise to series of “coordinated” ACs in which many compounds are involved in multiple cliffs [9]. Regardless of the AC criteria that are applied, greater than 90% of all ACs found in compound activity classes are formed in a coordinated manner [9]. AC coordination can be explored in network representations, in which nodes represent compounds and edges pairwise ACs. In such networks, coordinated ACs give rise to the formation of AC clusters of varying size and topology [9]. AC clusters have higher SAR information content than ACs studied individually but, their interactive analysis is arduous when clusters increase in size and their topologies become rather complex [10]. Therefore, attempts have been made to computationally extract SAR information from AC clusters, for example, by organizing them in index maps on the basis of different intra-cluster structural relationships [10] or by isolating sequences of AC compounds from clusters that follow a potency gradient [11]. These approaches help to dissect clusters selected from AC networks and isolate AC subsets, providing at least partial access to SAR information.

While AC networks are essential for the rationalization and exploration of coordinated ACs, the interpretability of complex networks is limited. Difficulties in interpreting complex AC networks hinder SAR exploration on the basis of AC clusters. Therefore, we have developed a network variant that reduces complexity and provides immediate access to SAR information, as reported herein.

## Materials and methods

### Compound activity classes

Activity classes for AC network analysis were extracted from ChEMBL release 26 [12]. Compounds directly interacting with human targets (target relationship type: “D”) at the highest assay confidence level (assay confidence score: 9) having equilibrium constants (K_i_ values) with exact “=” relationships as potency measurements were selected. If multiple measurements were available they were averaged, provided all potency values fell within the same order of magnitude; otherwise, the compound was disregarded. Table 1 summarizes the composition of three large activity classes used for AC network analysis.

**Table 1:** Activity classes

Target ID	Target name	No. CPDs	pK_i_ range	No. MMP-cliffs
259	Melanocortin receptor 4	1281	[3.65, 10.10]	426
244	Coagulation factor X	1641	[3.59, 11.40]	915
237	Kappa opioid receptor	1982	[4.09, 11.52]	987

For AC network analysis, three large activity classes were taken from ChEMBL. For each class, the ChEMBL target ID, target name, number of qualifying compounds (CPDs), their potency value (pK_i_) range, and the number of MMP-cliffs are reported.

### Compound decomposition

Systematic single-cut fragmentation of exocyclic single bonds was carried out using an algorithm for the generation of matched molecular pairs (MMPs) [13]. An MMP is defined as a pair of compounds that are only distinguished by a chemical modification at a single site [13]. During each fragmentation step two fragments per compound were obtained including a core and a substituent. In the core, a hydrogen atom was added to the substitution site. Size restrictions were applied to confine cores and substituents to those typically observed in analog series [14]. First, the number of non-hydrogen (heavy) atoms in the core was required to be at least twice as large as in the substituent. Second, the substituent fragment was restricted to at most 13 heavy atoms. Third, the size difference between exchanged substituents in an MMP was set to at most eight heavy atoms.

### Activity cliffs

For AC analysis, the MMP-cliff definition was used [14], which is tailored towards medicinal chemistry applications [6]. Accordingly, as AC criteria, two compounds from the same activity class are required to form a size-restricted MMP and have an at least 100-fold potency difference (ΔpK_i_ ≥ 2.0). By definition, MMP-cliffs contain a single substitution site.

### Matching molecular series

**Fig. 1:**
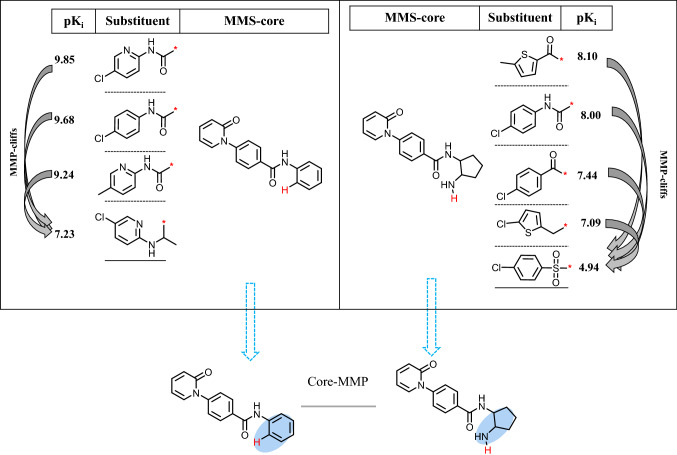
Structural relationships. Shown are two MMSs of coagulation factor X inhibitors that contain multiple MMP-cliffs (indicated by curved arrows) and form an MMSP. MMSs are represented as R-group tables including compound potency (pKi) values. Hydrogen atoms added to the substitution sites in the two MMS-cores are colored red. The core-MMP resulting from the second round of fragmentation that establishes the relationships between these MMSs is shown at the bottom. Substituents distinguishing between MMS-cores are shown on a blue background.

As an extension of MMP concept, matching molecular series (MMSs) were systematically extracted from all AC compounds. An MMS consists of two or more analogs that share the same core (MMS-core) and are only distinguished by substituents at a single site [15]. All identified MMS-cores were subjected to a second round of MMP fragmentation, as described above, to identify structurally analogous cores. Two MMS-cores were structurally analogous if they formed a core-MMP and the corresponding MMSs were the classified as an MMS-pair (MMSP). Figure 1 shows an exemplary MMSP.

### Networks

AC networks were generated in which nodes represent compounds and edges indicate the formation of pairwise MMP-cliffs [14]. Reduced AC networks were designed as detailed below. All network representations were drawn with Cytoscape [16].

## Results and discussion

### Network design principles

**Fig. 2:**
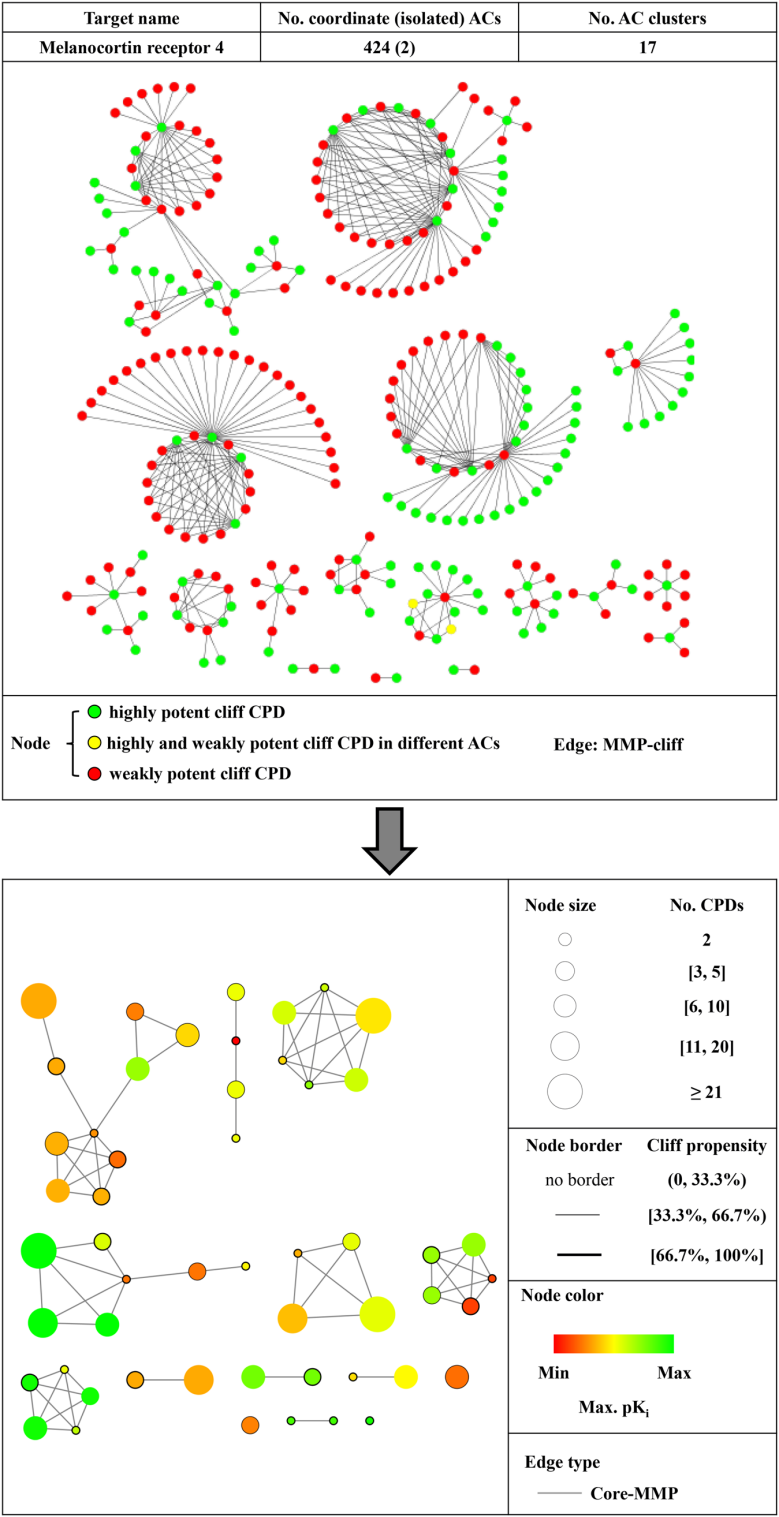
Activity cliff network representations. At the top, the AC network formed by melanocortin receptor 4 ligands is shown that contains 424 coordinated and two isolated MMP-cliffs. Nodes represent AC compounds and edges the formation of pairwise MMP-cliffs. Nodes are color-coded to distinguish three types of AC compounds: green, highly potent AC compound; red, weakly potent AC compound; yellow, highly/weakly potent compound in different ACs. The network reveals the formation of AC clusters of varying size and topology. At the bottom, the reduced network is displayed. Design principles, as discussed in the text, are summarized on the right. In the reduced network, nodes represent MMSs and edges pairwise MMSP relationships

AC networks such as the one shown in Fig. 2 (top) are essential for visualizing and rationalizing the coordinated formation of ACs. Moreover, individual clusters emerging in AC networks provide a basis for the extraction of SAR information. With a total of 426 ACs (including only two isolated ACs) organized in 17 clusters, the AC network for melanocortin receptor 4 ligands has moderate size and complexity and is interpretable. However, extracting SAR information from the three largest clusters is already difficult, if not impossible by interactive analysis, requiring the application of computational approaches [10, 11]. We note that the use of the MMP concept as a substructure-based similarity criterion for AC formation supports interpretability of the network structure because MMP relationships are clearly defined and select structural analogs modified at a single site as AC compounds. Moreover, extension of the MMP concept through the MMS formalism makes it possible to trace MMSs in AC clusters as a basis for series-centric SAR analysis [11]. However, tracing single or multiple MMSs in AC clusters does not simplify the network structure [11].

To enable interpretation of AC networks of increasing size and complexity and facilitate direct extraction of SAR information, we have developed an approach for the reduction of AC networks that employs the MMS formalism in different ways. Design principles for the simplified network are summarized in Fig. 2 (bottom). A central idea underlying the network reduction approach is transforming the entire cluster structure of the AC network into an array of MMSs and MMSPs. Thereby, all ACs are represented on the basis of MMSs and structurally related MMS-cores are identified. In the corresponding reduced network, each node represents an MMS comprising two or more analogs. The inclusion of compound pairs accounts for isolated ACs. Edges between nodes indicate MMSP relationships (in algorithmic terms, the formation of a core-MMP). Nodes are scaled in size according to the number of compounds per MMS and can be color-coded according to different potency characteristics (or other properties) such as the largest potency of MMS members. This color scheme accounts for the distribution of highly potent AC compounds across MMSs. AC information is also conveyed through node borders, the thickness of which reflects the AC propensity within MMSs. Propensity represents the percentage of all possible analog pairs that form an AC in a given MMS. By design, individual MMS clusters in the reduced network may combine multiple original AC clusters, but have simpler topologies and limited complexity. However, all AC information is retained and MMSs or MMSPs with high AC propensity can be readily identified and selected for further analysis.

### Reducing complex activity cliff networks

**Fig. 3:**
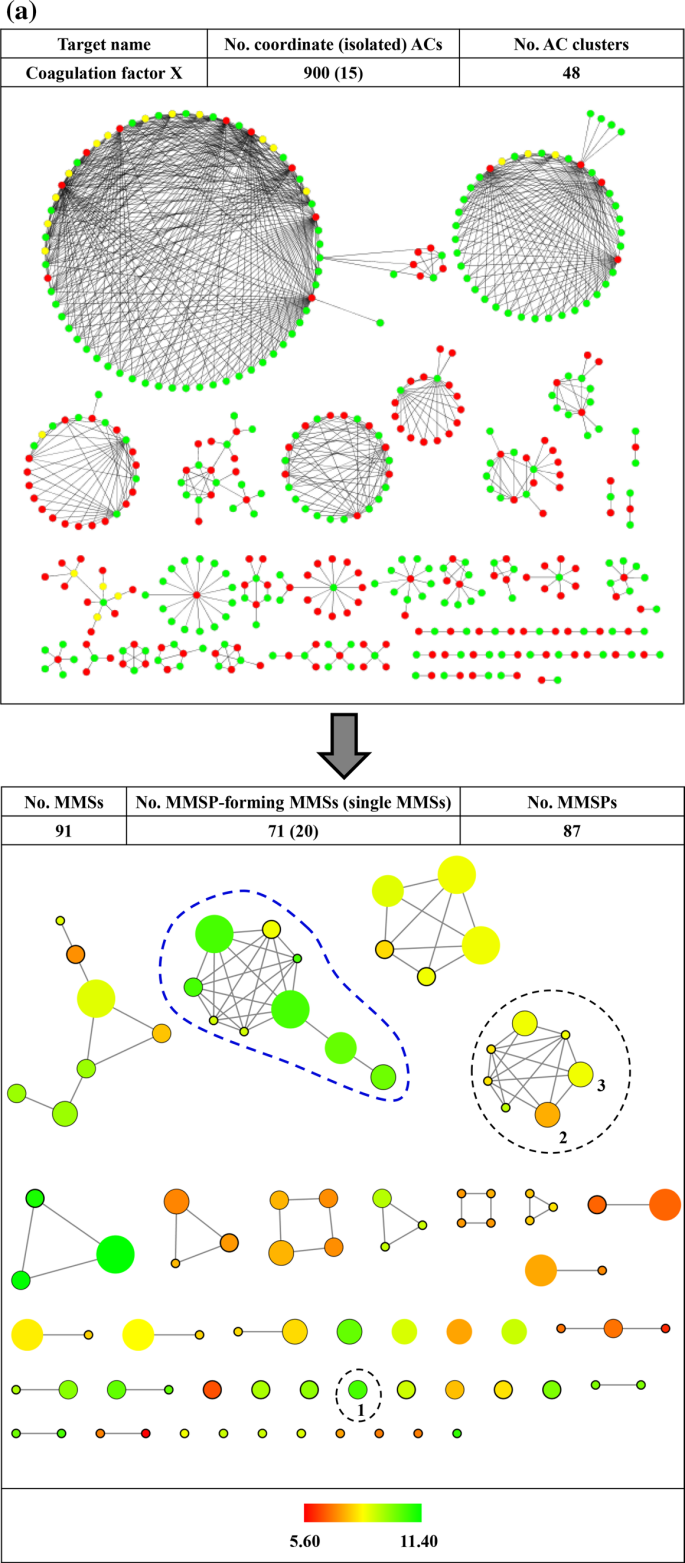

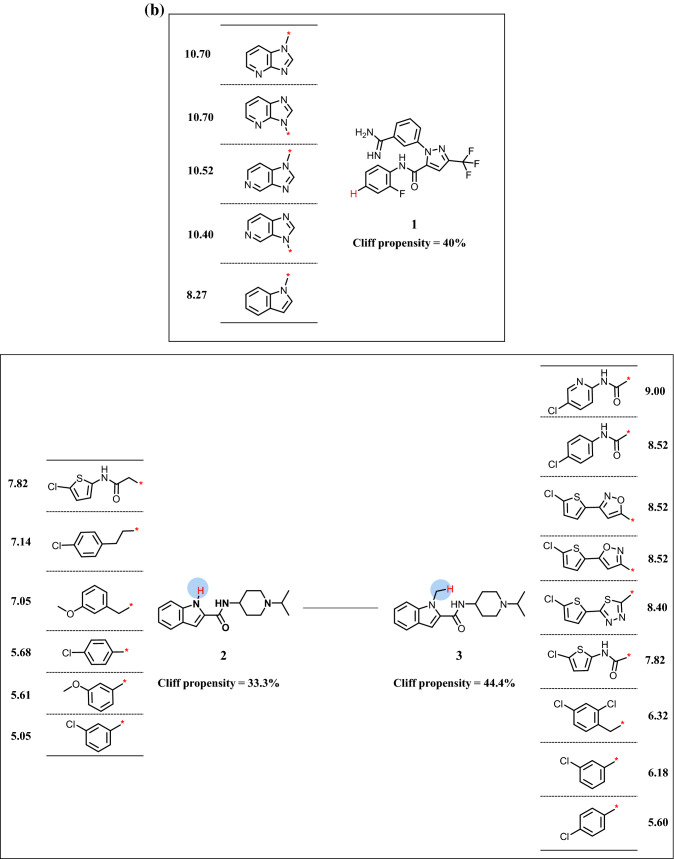
Activity cliff networks for coagulation factor X inhibitors. In **a**, the original AC network (top) and the reduced network (bottom) are displayed according to Fig. 2. Numbers at an encircled node and cluster mark an exemplary isolated MMS (1) and an MMSP (2/3), respectively. In **b**, R-group tables representing the isolated MMS (top) and MMSP (bottom) are shown.

**Fig. 4:**
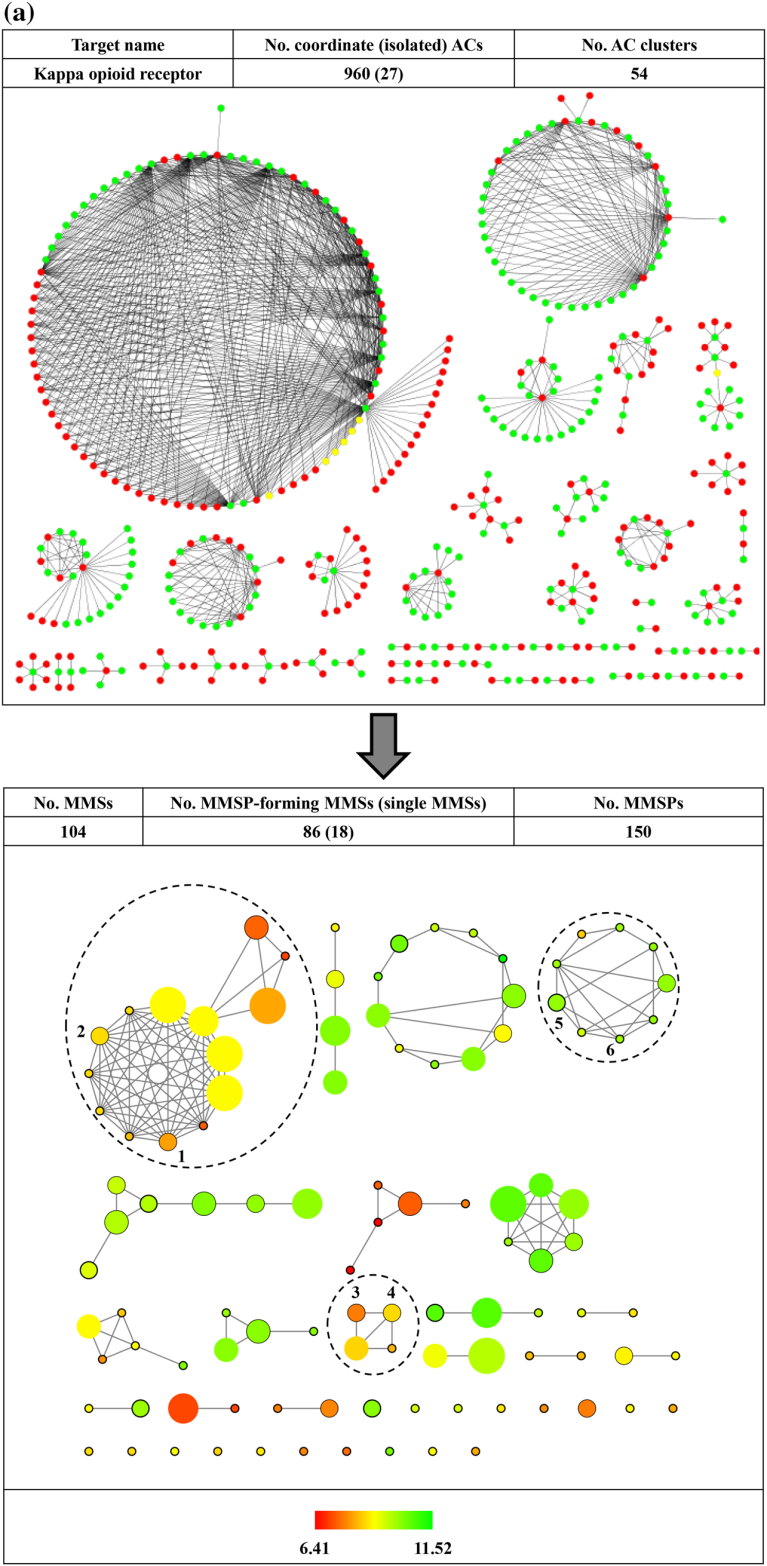

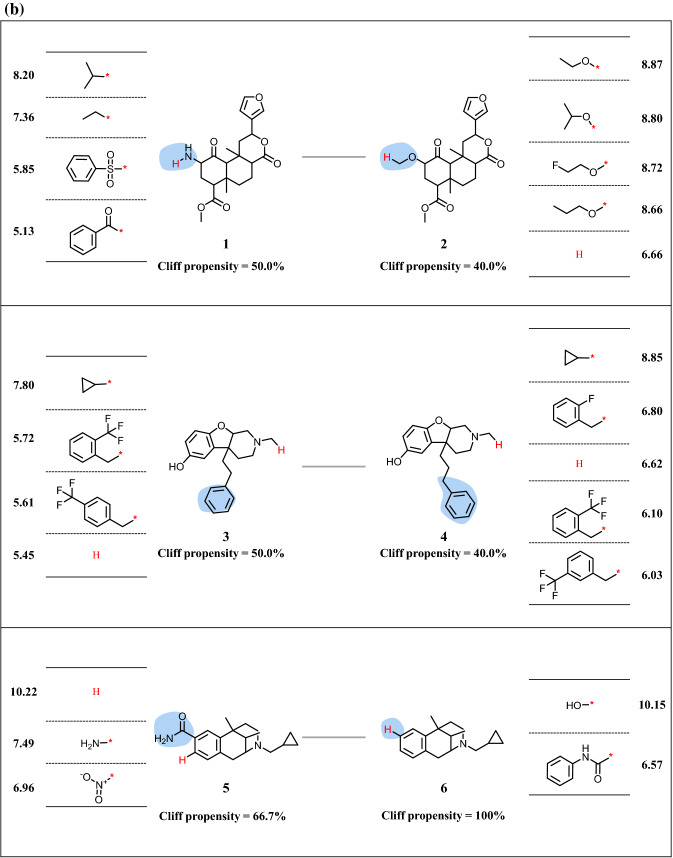
Activity cliff networks for kappa opioid receptor. In **a** the original AC network (top) and the reduced network (bottom) are displayed according to Fig. 2. Numbers at encircled clusters mark three exemplary MMSPs (1/2, 3/4, and 5/6). In **b** R-group tables representing the three MMSPs are shown

The utility of reduced networks becomes immediately evident when AC networks of increasing size and complexity are considered such as the example in Fig. 3a. The network at the top consists of 915 ACs (including only 15 isolated ACs) and contains several densely connected spherical clusters. The two largest AC clusters are essentially impossible to analyze interactively. By contrast, the reduced network at the bottom is immediately interpretable. It consists of 91 MSSs including 71 that form a total of 87 MMSPs. In addition, there are 20 single MSSs. In the reduced network, the largest AC cluster (with 363 ACs) from the original network is mostly (96%) represented by the MMS cluster encircled using a blue dashed line. It can be seen that this cluster combines nine MMSs of greatly varying size that contain highly potent cliff compounds. Seven of the nine MMSs are densely connected including the two largest and the smallest ones. The remaining two MMSs only form one or two pairs including a medium size MMS with multiple ACs. In contrast to the original AC network, the reduced network can be easily navigated including the largest clusters. Another example is shown in Fig. 4a. Here, the AC network of kappa opioid receptor ligands (top) comprises 987 ACs that are organized in 54 clusters, the largest of which dominates the network view. In the reduced network (bottom), this very large and densely connected cluster (with 493 ACs) is exclusively represented by the encircled MMS cluster at the upper left (containing MMSP 1/2). Other clusters in the reduced network have simple topologies and are straightforward to analyze.

### Extracting SAR information from reduced networks

A key feature of reduced networks is that individual MMSs or MMSPs of interest can be easily selected and represented in standard R-group tables. These tables are most popular in medicinal chemistry for the representation of analog series and provide immediate access to SAR information including ACs formed within the MMSs. Examples are shown in Figs. 3b and 4b. Compared to original AC networks, extraction of SAR information from reduced networks is greatly simplified. Notably, generating R-group tables from MMSPs, as shown in Figs. 3b and 4b, further supports SAR analysis compared to single MMSs. This is the case because MMS-cores of MMSPs are structurally analogous by design. Since these cores are algorithmically generated for large-scale AC analysis, they should always be compared from a chemical perspective when individual MMSs are considered. In some instances, algorithmically defined cores might be chemically sufficiently similar such that the R-group tables of the MMSP can be jointly analyzed or even combined. For example, this would be the case for the MMSP in Fig. 3b. In other instances, cores might be chemically distinct -although they are structurally analogous- likely giving rise to different SAR characteristics exhibited by related MMSs. Examples are provided in Fig. 4b. Since these MMSs from reduced networks contain ACs, they likely reveal SAR determinants for related yet distinct series. The reduced networks provide many opportunities for comparing SARs encoded by MMSPs on the basis of their R-group tables, which benefits SAR exploration from a medicinal chemistry perspective.

## Conclusions

The vast majority of ACs are formed in a coordinated manner. For their analysis, network representations play a central role. In AC networks, coordinated ACs centred on different analog series emerge as disjoint clusters of different composition and varying topology. These AC clusters become a primary focal point for SAR exploration. However, with increasing size and complexity, AC networks become difficult to navigate and clusters hard to analyze interactively. Accordingly, there is a need for making coordinated ACs and the information they provide available in a format that is readily interpretable. We have reasoned that network reduction might be suitable for this purpose, provided that AC information could be fully retained. Therefore, in this work, we have introduced an approach for the generation of simplified AC networks that is conceptually based upon the MMS formalism and the assessment of structural relationships between MMSs. In reduced networks, resulting MMSPs and individual MMSs resolve the original AC cluster structure and replace it with a higher-level structural organization scheme, which results in simplified network views and ensures interpretability. This represent a key aspect of the design strategy. As shown herein, original and reduced networks can be analyzed side-by-side, providing complementary views. Moreover, from reduced networks, MMSs and MMSPs can be easily selected and represented as R-group tables that reveal ACs and SAR information. This is another key feature of the approach. Presenting analog series from simplified networks in the form of R-group tables enables SAR analysis from a medicinal chemistry perspective, without requiring further computational input, and hence supports practical applications. In our proof-of-concept study, representative activity classes and AC populations have been investigated to demonstrate the utility of the approach. Reduced networks have been generated for many more activity classes, consistently enabling interpretation of AC clusters and SAR analysis on the basis of R-group tables. We also note that reduced network representations will not replace original AC networks, but are designed to aid in their analysis through the generation of complementary simplified views. AC networks remain important tools for globally visualizing the coordinated formation of ACs and comparing AC populations originating from different compound data sets. However, reduced networks will be essential for detailed analysis of large AC clusters with complex topologies.

## Electronic supplementary material

Below is the link to the electronic supplementary material.

Electronic supplementary material 1 (PNG 288 kb)

Electronic supplementary material 2 (PNG 986 kb)

Electronic supplementary material 3 (PNG 1458 kb)

Electronic supplementary material 4 (PNG 326 kb)

Electronic supplementary material 5 (PNG 1736 kb)

Electronic supplementary material 6 (PNG 309 kb)
