# Supplementary figures and images for: Simplified activity cliff network representations with high interpretability and immediate access to SAR information
Source: J Comput Aided Mol Des. 2020 Jun 5;34(9):943–52. doi: 10.1007/s10822-020-00319-9 (PMC7367913; doi:10.1007/s10822-020-00319-9)

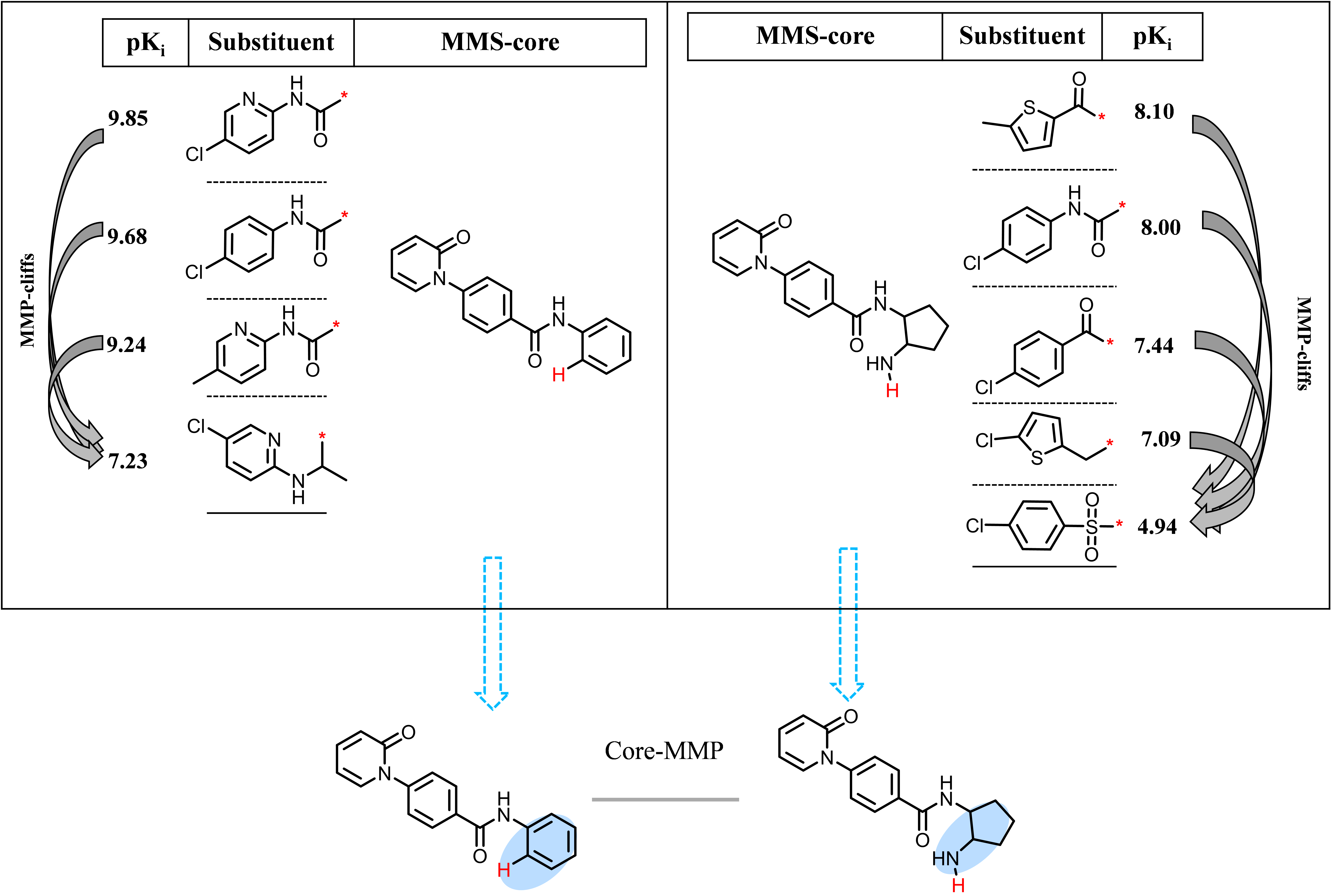

Supplement: Supplementary file 1 — Electronic supplementary material 1 (PNG 288 kb) [file 10822_2020_319_MOESM1_ESM.png]

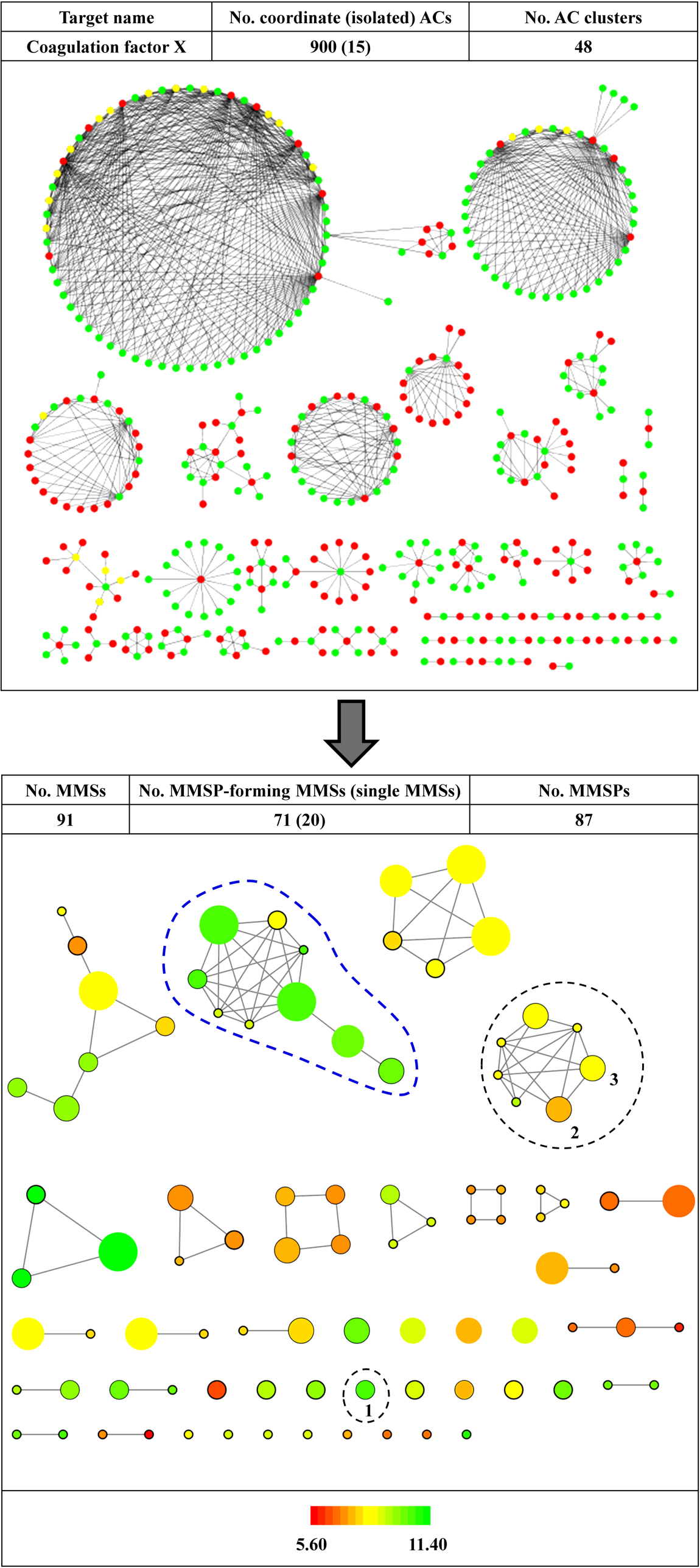

Supplement: Supplementary file 3 — Electronic supplementary material 3 (PNG 1458 kb) [file 10822_2020_319_MOESM3_ESM.png]

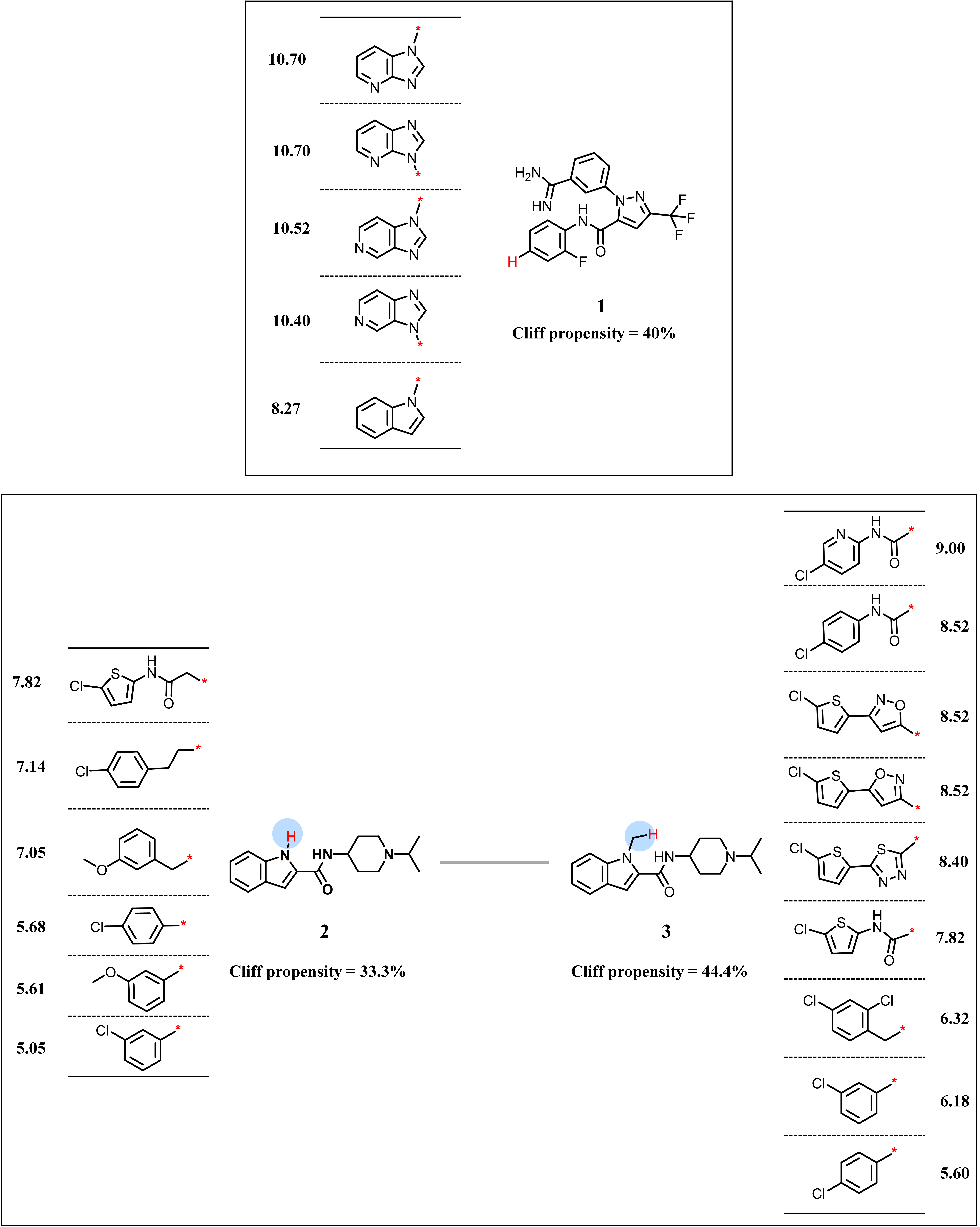

Supplement: Supplementary file 4 — Electronic supplementary material 4 (PNG 326 kb) [file 10822_2020_319_MOESM4_ESM.png]

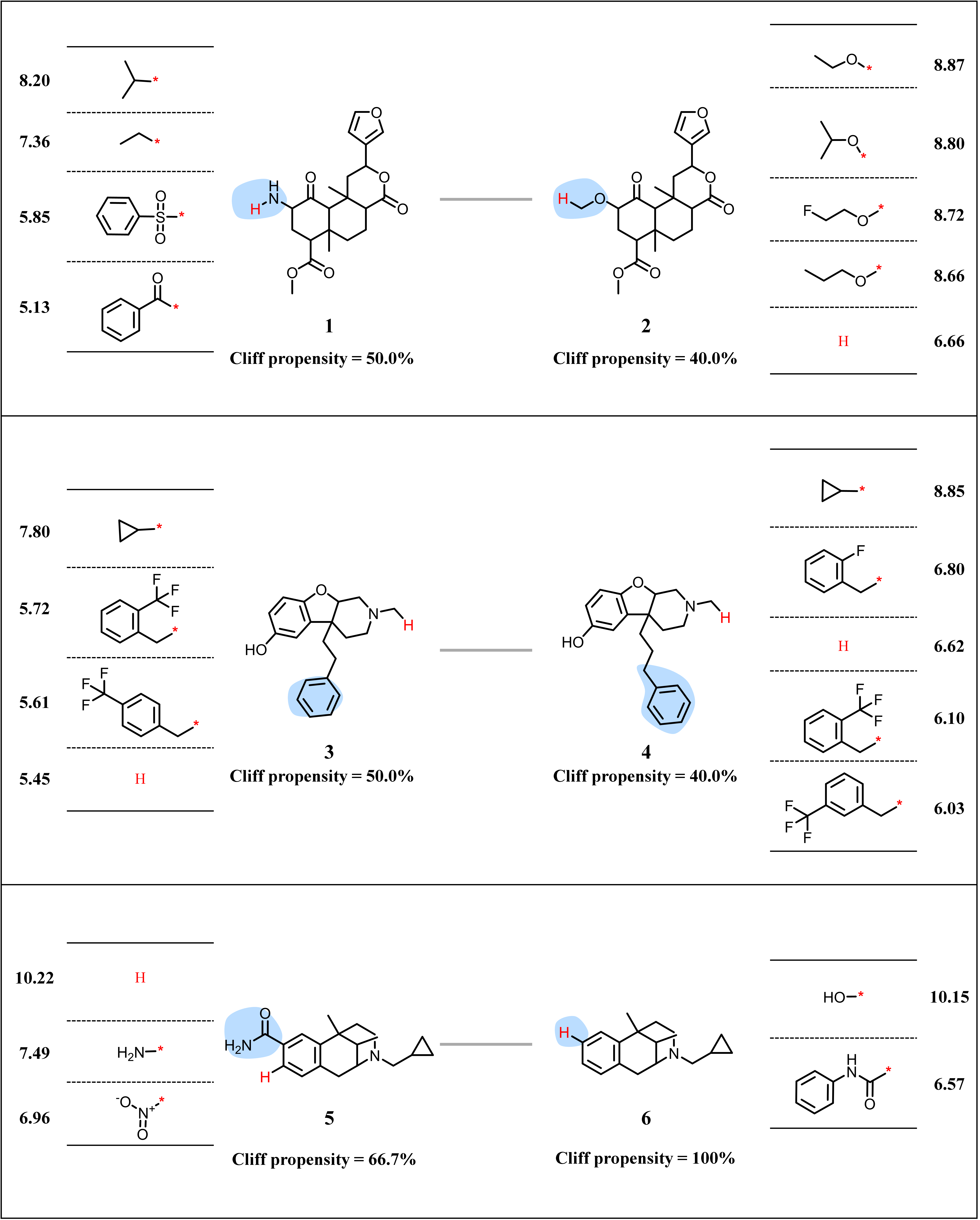

Supplement: Supplementary file 6 — Electronic supplementary material 6 (PNG 309 kb) [file 10822_2020_319_MOESM6_ESM.png]
